# Serotonergic stimulation induces nerve growth and promotes visual learning via posterior eye grafts in a vertebrate model of induced sensory plasticity

**DOI:** 10.1038/s41536-017-0012-5

**Published:** 2017-03-30

**Authors:** Douglas J. Blackiston, Khanh Vien, Michael Levin

**Affiliations:** Allen Discovery Center, Tufts University, 200 Boston Avenue, Suite 4600, Medford, MA 02155 USA

## Abstract

The major goal of regenerative medicine is to repair damaged tissues and organ systems, thereby restoring their native functions in the host. Control of innervation by re-grown or implanted structures, and integration of the nascent nerves into behavioral/cognitive programs of the host, remains a critical barrier. In the case of sensory organs, this is particularly true, as afferent neurons must form connections with the host to communicate auditory, visual, and tactile information. *Xenopus* embryos and tadpoles are powerful models for such studies, as grafting techniques allow for the creation of eyes and other sensory structures along the body axis, and the behavior of the resulting organism can be quantitatively analyzed. Previous work has demonstrated that ectopic eyes could be grafted in blinded tadpoles, allowing some of the animals to learn in a simple light-preference assay. Here, we show that it is possible to improve the efficiency of the process in the context of a novel image-forming vision assay, using a drug already approved for human use. Innervation of the host by ectopic eyes can be increased by targeting a serotonergic signaling mechanism: grafts treated with a 5-HT_1B/D_ agonist strongly innervate the recipient compared with untreated grafts, without large-scale disruption of the host nervous system. Blind animals possessing eye grafts with the augmented innervation demonstrate increased performance over untreated siblings in wavelength-based learning assays. Furthermore, treated animals also exhibit enhanced visual pattern recognition, suggesting that the increased innervation in response to 5-HT_1B/D_ activation leads to enhanced functional integration of the ectopic organ with the host central nervous system and behavioral programs. These data establish a model system and reveal a new roadmap using small molecule neurotransmitter drugs to augment innervation, integration, and function of transplanted heterologous organs in regenerative medicine.

## Sensory organs: drug induces nerve growth for ectopic eye transplant

A migraine drug that modulates neurotransmitter signaling can boost the neural connections of eyes grafted onto tadpoles to enhance vision. Michael Levin and colleagues from Tufts University in Medford, MA, USA, built on previous work from their lab showing that eyes could be attached along the body axis of blind tadpoles, allowing the developing frogs to distinguish between light and dark. Following the surgery, the researchers have now added the drug zolmitriptan, an activator of serotonin receptors, and the tadpoles formed many more neural connections that sprouted from their ectopic eyes. These animals performed better in visual-learning and pattern-movement tests than control tadpoles that did not get the drug. The findings suggest that drugs used to treat neurological and psychiatric diseases could be repurposed to augment the innervation, integration, and function of organs transplanted in regenerative therapies.

## Introduction

The future of regenerative medicine will surely involve the transplantation of a wide variety of bioengineered structures, from hybrid artificial constructs to stem cell-derived organoids grown in vitro. A key aspect of this research program is the establishment of neural connections between the host central nervous system (CNS) and the implanted tissue, to restore normal function or to provide augmented sensory/motor capability to patients. In addition to the biomedical aspects, this research provides an unprecedented opportunity for basic research into the relationship between the brain and body, and the evolution of plasticity of cognitive programs in the face of altered bodyplans. Novel models in which the ability of the brain to make use of ectopic organs must be developed in vertebrate animal species that are tractable to molecular, physiological, and behavioral assays. Such platforms would enable the study of the inherent plasticity that might be exploited for sensory repair or even enhancement, and the evaluation of potential treatments to improve function in such contexts.

The resolution with which implanted structures must communicate with the host’s CNS remains poorly understood. Conventional theory suggests that specific, direct communication between sensory structures and their corresponding processing centers within the brain is required for function. However, sensory substitution studies using human–machine interfaces have revealed a striking plasticity of the CNS to interpret sensory data presented at novel locations. The majority of this work has focused on tactile-vision substitution devices, which transmit wavelength information from a camera to electrical or vibratory stimulation via a small array that the patent places on their body (dermal stimulation) or tongue (where the saliva allows less current to deliver a similar stimulus).^1^ Blind individuals using these devices report imaging formation consistent with vision,^2^ and positron emission tomography (PET) imaging of the brains of users reveal activity within the visual centers of the occipital lobe.^3, 4^ This tactile vision involved more than simple object recognition: subjects were able to time their actions to intercept a ball rolling off of a surface and coordinate eye-hand movements in three-dimensional space.^5^ Beyond vision, similar devices have been used in a variety of other sensory modalities, including auditory–visual substitution,^6, 7^ tactile-vestibular (balance) substitution,^8^ and a sensory augmentation belt that signals the direction of magnetic north.^9^

All of these devices highlight the adaptive nature and remarkable plasticity of the brain:body system. However, the cross-modal functionality revealed by these studies is limited in comparison with the goal of fully integrated biological organs such as regenerated or transplanted eyes. Progress in the sophistication of 3D culture methods allows for the in vitro creation of morphologically complete mouse and human optic cups,^10, 11^ and a major goal of regenerative medicine is to one day implant these structures in human patients, thereby restoring the function of damaged or missing sensory systems. However, what remains unknown are the necessary signals that could be used to promote sufficient afferent innervation between the implant and host and whether the host brain could properly interpret sensory information from structures that were added long after embryogenesis.

A number of secreted molecules involved in axon growth cone extension and pathfinding have been identified using in vitro developmental models and neuronal culture systems. In the case of the visual system these include sonic hedgehog as well as Ephrin, Netrin, Semaphorin, and Slit family members, which coordinate the exit of retinal ganglion cell axons from the optic cup to visual-processing centers within vertebrate brain. In addition to these well-studied factors, serotonin has also been identified as a pathfinding cue during neural development. In invertebrates, cultured *Helisomia* snail neurons show growth changes response to extracellular serotonin.^12^ In vertebrates, serotonin has been shown to reverse the effect of netrin-1 from attraction to repulsion when both were presented to cultured mouse thalamocortical axons^13^; in addition, serotonin levels have been shown to alter segregation of retinal ganglion projections within the dorsal lateral geniculate nucleus and superior colliculus in vivo.^14^

*Xenopus laevis* represents an ideal model to investigate graft and implant innervation. Specifically, cut and paste experiments allow the generation of eyes and ears at any position along the body axis of developing tadpoles^15–21^: in both cases, afferent innervation between the graft and host CNS was observed, even when the sensory structures were positioned far from their native locations. Remarkably, behavioral assays showed that such grafted eyes were capable of conferring light-sensing ability to blinded tadpoles. However, good performance in visual assays was infrequent because most grafts failed to innervate the host. We have been testing strategies for inducing innervation of ectopic structures, focusing on bioelectric properties of the host and the neurotransmitter molecules downstream of voltage-dependent signaling.^22–25^ Here, we demonstrate that strong innervation can be induced for eye grafts by exposure to a 5-HT_1B/D_ agonist, without significantly altering elements of the host CNS. Moreover, this induced innervation, causing animals receiving treated grafts to perform significantly better in wavelength-discrimination tasks than animals receiving untreated grafts. We show that the function of ectopic eyes in otherwise blind animals is not only for light sensing, but to confer performance in a novel behavior assay that requires image-forming vision (pattern detection). Taken together, these data demonstrate that organ grafts are capable of providing sensory information to a vertebrate host, even when located far from the brain, and they reveal a novel strategy for promoting innervation of implanted sensory structures using a pharmacological reagent already approved for human use.

## Results

### 5-HT_1B/D_ activation induces innervation of transplanted tissue following eye graft in *Xenopus* embryos

**Fig. 1:**
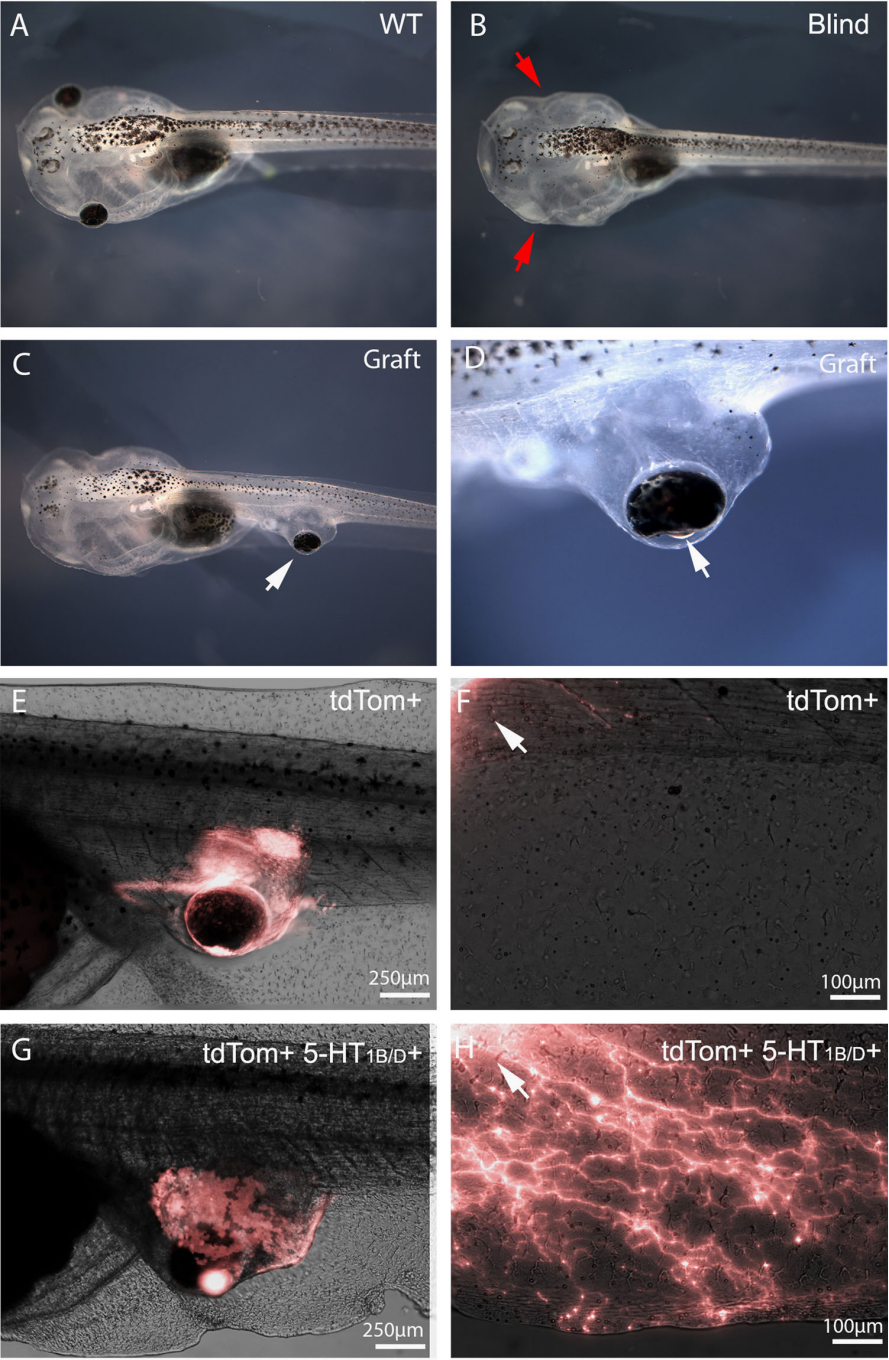
Serotonin receptor 1B/D activation increases afferent innervation of eye grafts in *Xenopus* tadpoles. **a** Wild-type animals possess fully developed eyes by stage 47. **b** Microsurgery performed on stage 34 animals to remove developing eye tissue leads to blind animals that are otherwise healthy. **c** Grafts of eye primordia transferred from stage 24 donors to recipients result in ectopic eyes forming at the site of the graft. **d** Eyes forming from grafts display proper proximal-distal orientation with a clearly visible lens (*arrow*) facing outward from the body. **e** Grafts between tdTomato labeled donors and non-labeled recipients allow donor tissue to be visualized at later stages. **f** Higher magnification of the region posterior to the graft (*white arrow*) reveals little innervation of the host, with few if any neurites present in the fin or trunk region of the animal. **g** Treatment of grafts with the 5-HT_1B/D_ activator Zolmitriptan does not alter the morphology of the ectopic eye. **h** Higher magnification of the region posterior to the graft (*white arrow*) reveals extensive afferent innervation in response to the treatment, with neurites present throughout the fin and trunk of the tadpole

In an effort to identify methods to increase the innervation of implanted sensory structures, we utilized an eye primordia graft method developed in *Xenopus laevis*, which results in morphologically complete eyes developing at the site of the graft. Grafts were performed embryonically between tdTomato mRNA injected donors and untreated recipients at Neiuwkoop and Faber stage 24, which allowed afferent innervation to be visualized under fluorescence. In addition, microsurgery at stage 34 could be performed to create blinded animals (compare Fig. 1a, b). Grafts were positioned caudally, which resulted in an ectopic eye forming in the posterior of the animal along the trunk of the tail (Fig. 1c). Induced eyes were morphologically similar to native eyes in both proximal/distal orientation and size, and vasculature could be seen carrying blood to the structure (Fig. 1d).

Donor tissue and innervation could be observed through fluorescence of the injected tdTomato protein (Fig. 1e) and imaging of the ventral fin posterior to the graft revealed little innervation of the host (Fig. 1f). Previous work that promoted graft innervation through modulation of host cells’ bioelectrical properties showed that innervation was induced by extracellular 5-HT exposure and could be blocked by inhibition of 5-HT receptor 1 family members.^24^ Based on these prior data identifying serotonergic signaling downstream of somatic depolarization,^24, 25^ we hypothesized that activation of these receptors should promote neurite outgrowth. Treatment of grafted animals with 50 µM of the 5-HT_1B/D_ activator Zolmitriptan following surgery had a dramatic effect on innervation: 40% of treated animals (*n* = 38) displayed a hyper-innervated phenotype with labeled neurites present throughout the ventral fin and trunk of the animal (Fig. 1g, h).

**Fig. 2:**
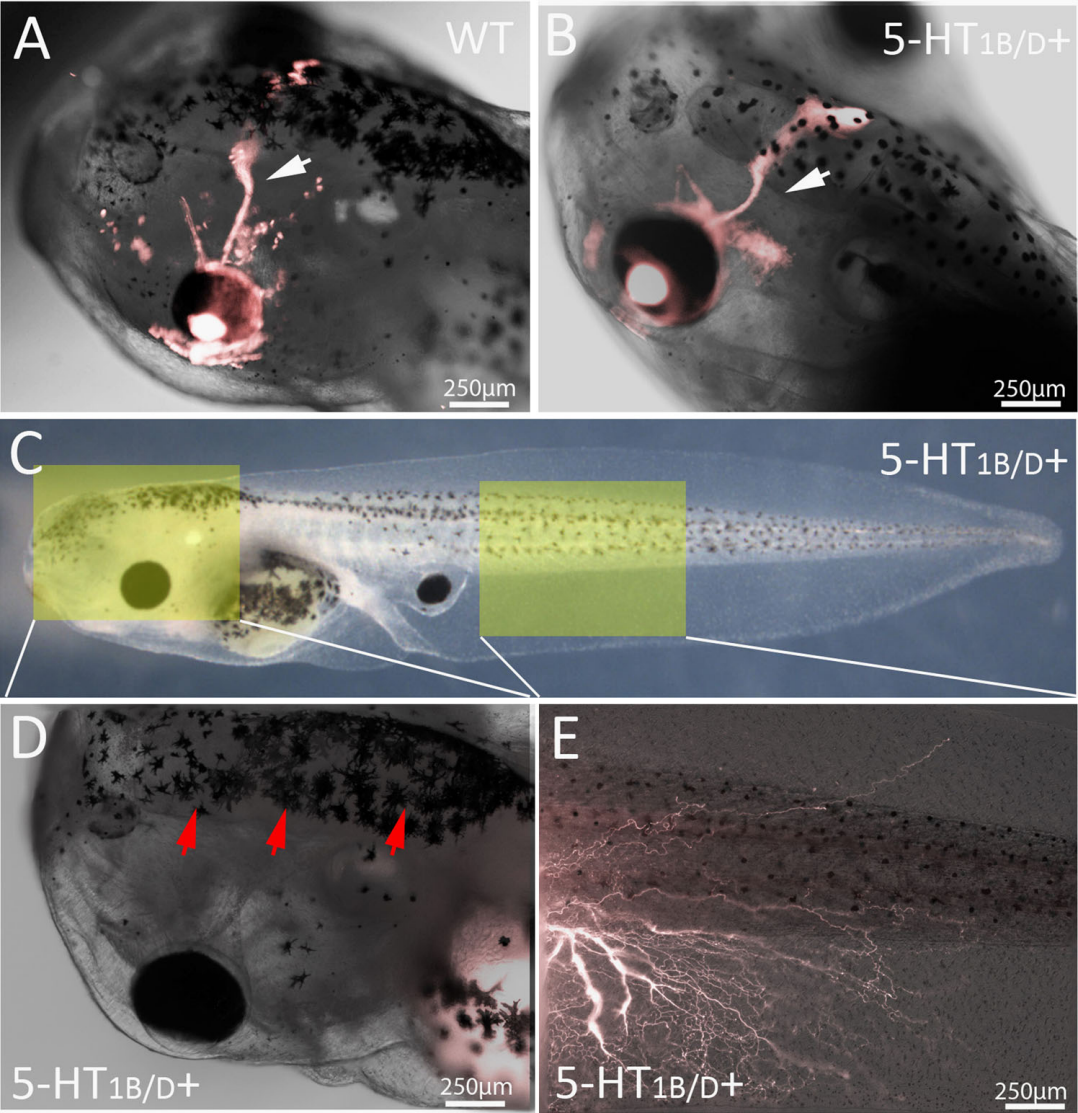
5-HT_1B/D_-induced innervation is location-specific. **a** When developing eye tissue is removed in stage 24 embryos and replaced with labeled donor tissue of the same type, optic nerves are visualized (*white arrow*) crossing to the contralateral side of the brain and penetrating at the optic tecta. **b** Grafts at correct developmental locations do not change their innervation in response to 5-HT_1B/D_ activation. **c** Animals with posterior grafts were visualized under fluorescence to determine if neurites reached the host brain. **d** Signal was never observed in the host brain (*red arrows*), **e** even when innervation was observed throughout the trunk and fin region of the animal (*n* = 20). *Yellow boxes* in (**c**) indicate location imaged at high magnification in (**d**) and (**e**)

To determine whether 5-HT_1B/D_ activation altered innervation of eyes in their native locations, developing eye tissue was removed from stage 24 wild-type animals and replaced with labeled donor tissue from the same location. In untreated animals, grafts produce labeled optic nerves which migrate ventrally below the brain before penetrating the contralateral side at the location of the optic tecta, as in the endogenous pattern (Fig. 2a). Animals receiving rostral grafts in the presence of 5-HT_1B/D_ activation demonstrate the normal innervation outcomes, sending specific, targeted projections to the brain rather than the broad, distributed innervation observed in caudal grafts (Fig. 2b). Thus, sham operations do not induce the hyperinnervated phenotype.

As innervation from such grafts was easily visible in the brain, posterior transplants were then specifically examined to determine if any of ectopic neurites were present in the brain of the host. In no case did we detect labeled neurons in brain of animals receiving posterior grafts (*n* = 20), even when strong innervation was present throughout the trunk and fin of the animal (Fig. 2c–e). We conclude that posterior grafts do not innervate directly into the brain.

**Fig. 3:**
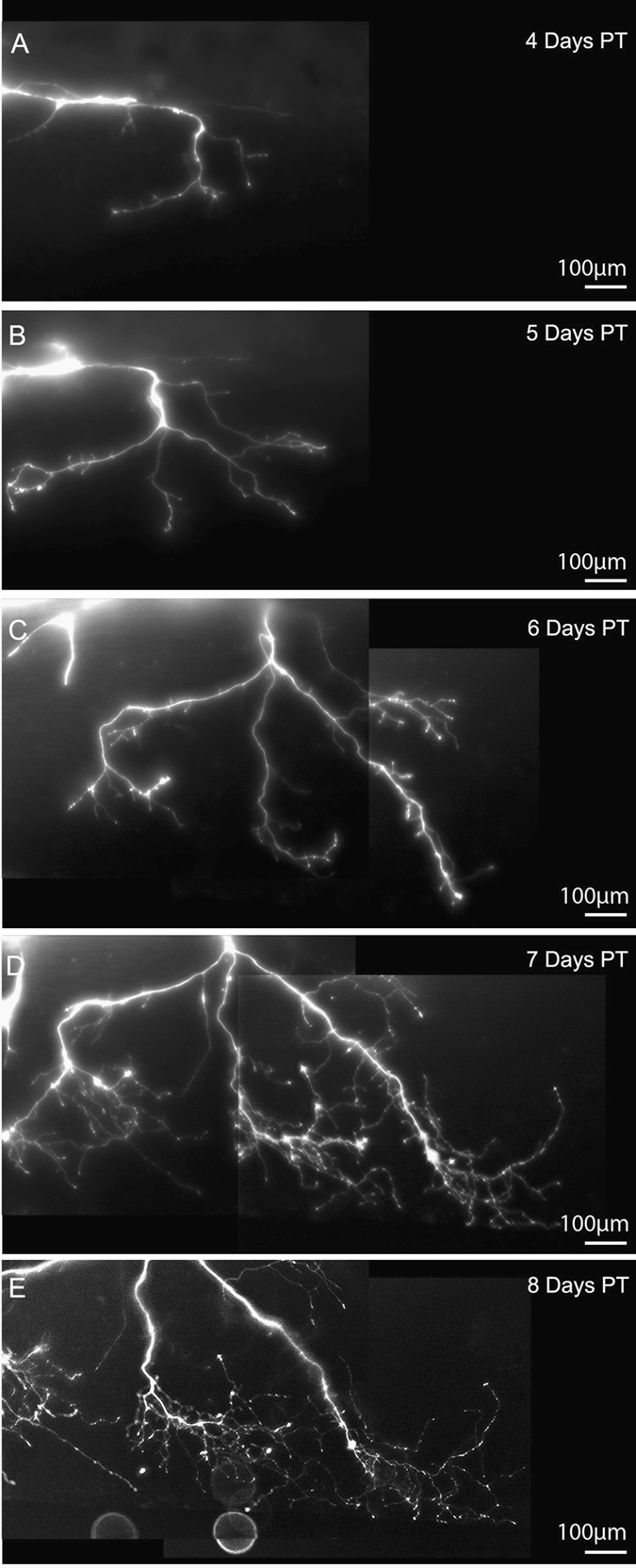
Time-lapse imaging of 5-HT_1B/D_-activated eye grafts reveals continued innervation and branching following treatment. **a** tdTomato labeled donor tissue was first observed innervating the host 4 days post-transplant. By 5 and 6 days post-transplant (**b**, **c**), neurites began to arborize and cover the ventral fin of the host. Arborization continued through days 7 and 8 (**d**, **e**), at which time high-branched neurites were present throughout the posterior of the recipient

To examine the timing of neurite outgrowth, animals were imaged daily for 8 days following surgery. No labeled axons were observed during the initial 3 days following surgery, but labeled tissue was clearly visible on day 4 (Fig. 3a). After this period, continued neurite elongation and branching was observed through the remainder of the experiment (Fig. 3b–e), with donor tissue innervating much of the ventral fin and trunk of the host. We conclude that a drastic hyper-innervation of transplants could be induced by Zolmitriptan exposure without toxic side effects.

### 5-HT_1B/D_ activation does not disrupt native innervation in the host

**Fig. 4:**
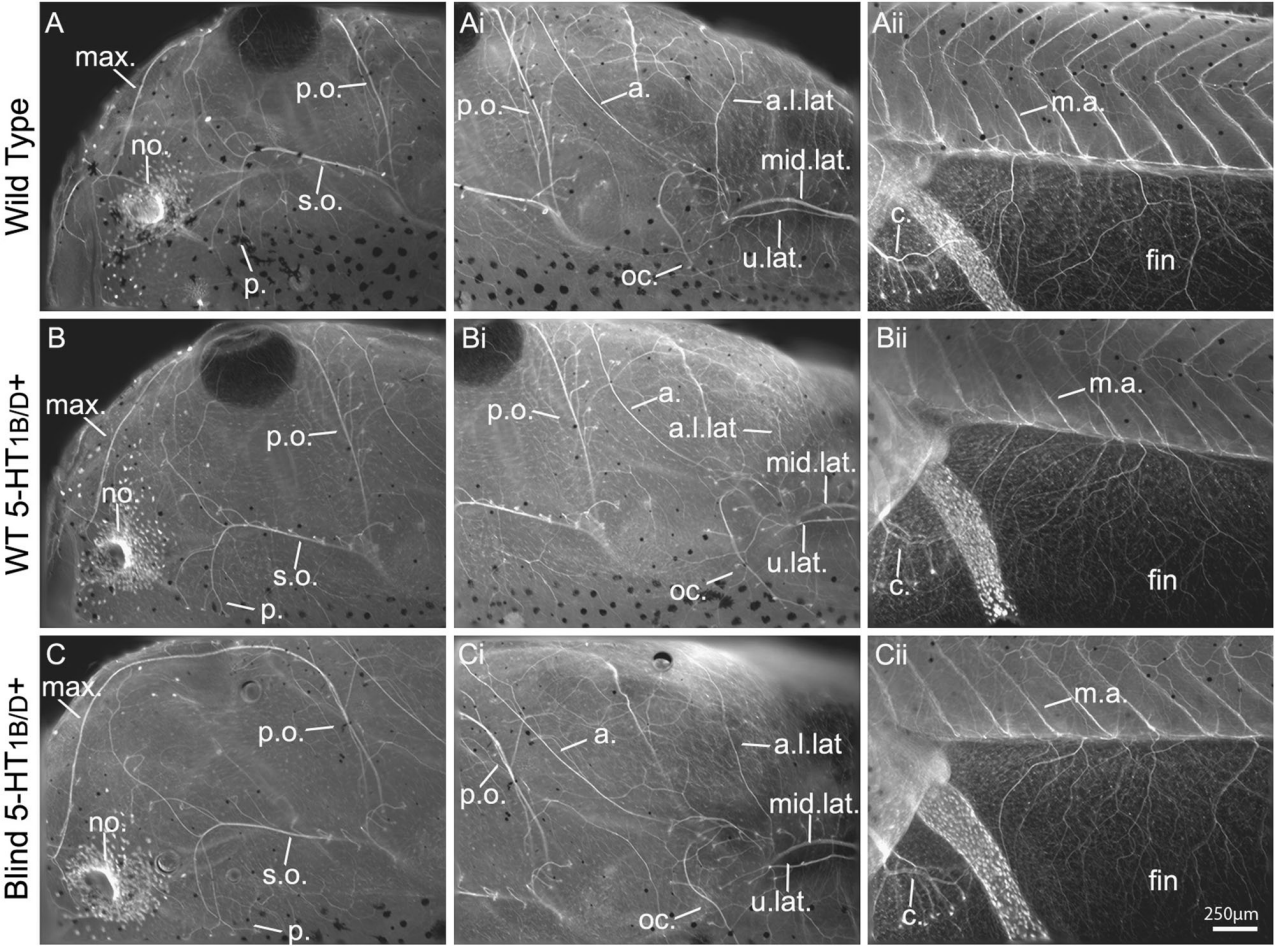
5-HT_1B/D_ activation does not alter host innervation. **a**i–iii Immunohistochemistry against acetylated-tubulin allowed visualization of lateral line components, nostrils, and motor axons. When comparing wild-type animals to 5-HT_1B/D_-activated (**b**i–iii) or 5-HT_1B/D_-activated blind animals (**c**i–iii), no differences were observed between treatments. *a*. aortic lateral line, *a.l.lat* anterior lower lateral line, *c.* caudal lateral line, *m.a.* motor axon, *max.* maxillary lateral line, *mid.lat.* middle lateral line, *o.c*. occipital lateral line, *no.* nostril, *p.* parietal lateral line, *p.o.* post-orbital lateral line, *s.o.* supra-orbital lateral line, *u.lat.* upper lateral line

The massive increase in graft innervation after 5-HT_1B/D_ receptor activation could be expected to be part of a universal neural response, which would disrupt the host nervous system. To test this hypothesis, we examined the anatomy of another sensory system, the host’s lateral line, in the presence or absence of 5-HT_1B/D_ activation. Using immunohistochemistry against the neuronal marker acetylated-tubulin, we also examined whether blinding animals caused a large-scale disruption to this system, as any perturbations could alter future behavioral trials. Immunohistochemical detection of neurons in wild-type animals revealed the stereotypic anatomy of lateral line elements in the anterior of the animal, with the post-orbital, maxillary, supra-orbital, and parietal lateral line being clearly defined (Fig. 4a). In addition, just anterior to the gut, the anterior lower, upper, middle, and occipital lateral line was visible (Fig. 4ai). Finally, in lateral images of the ventral tail fin around the anus of the animal, the caudal lateral line was visualized its standard branching phenotype (Fig. 4aii).

In animals raised in 50 µM of the 5-HT_1B/D_ agonist Zolmitriptan from stages 24–46, the lateral line elements were unperturbed, showing the same anatomy and branching patterns as that of untreated animals (Fig. 4bi–iii). Further, lateral line anatomy was also visualized in blinded animals to determine if the removal of native eyes altered host innervation in the presence of 5-HT_1B/D_ activation. No differences were noted between the lateral line elements of these animals compared with wild types, other than the connection between the post-orbital and maxillary lateral line being visible, as it is normally obstructed by the presence of the eye (Fig. 4ci–iii). The nostrils of the animals in all three treatments were also similar with regard to morphology and position (compare “no”, Fig. 4a, b, c). In the posterior, the motor axons located between somite boundaries maintained their characteristic chevron pattern, regardless of treatment (“m.a.” in Fig. 4aii, bii, cii). Finally, the ventral fin, which is the site of eye primordia grafts, did not show aberrant innervation by the lateral line and was devoid of ectopic innervation in the absence of donor tissue (“fin” in Fig. 4aii, bii, cii). Taken together, these data indicate that while grafted eye primordia respond to the 5-HT_1B/D_ agonist, the host nervous system is not detectably altered when these receptors are activated. These findings highlight the ability to functionally dissociate responses between the host’s own innervation and ectopic structures.

### Animals with innervated grafts demonstrate learning in an automated visual assay

**Fig. 5:**
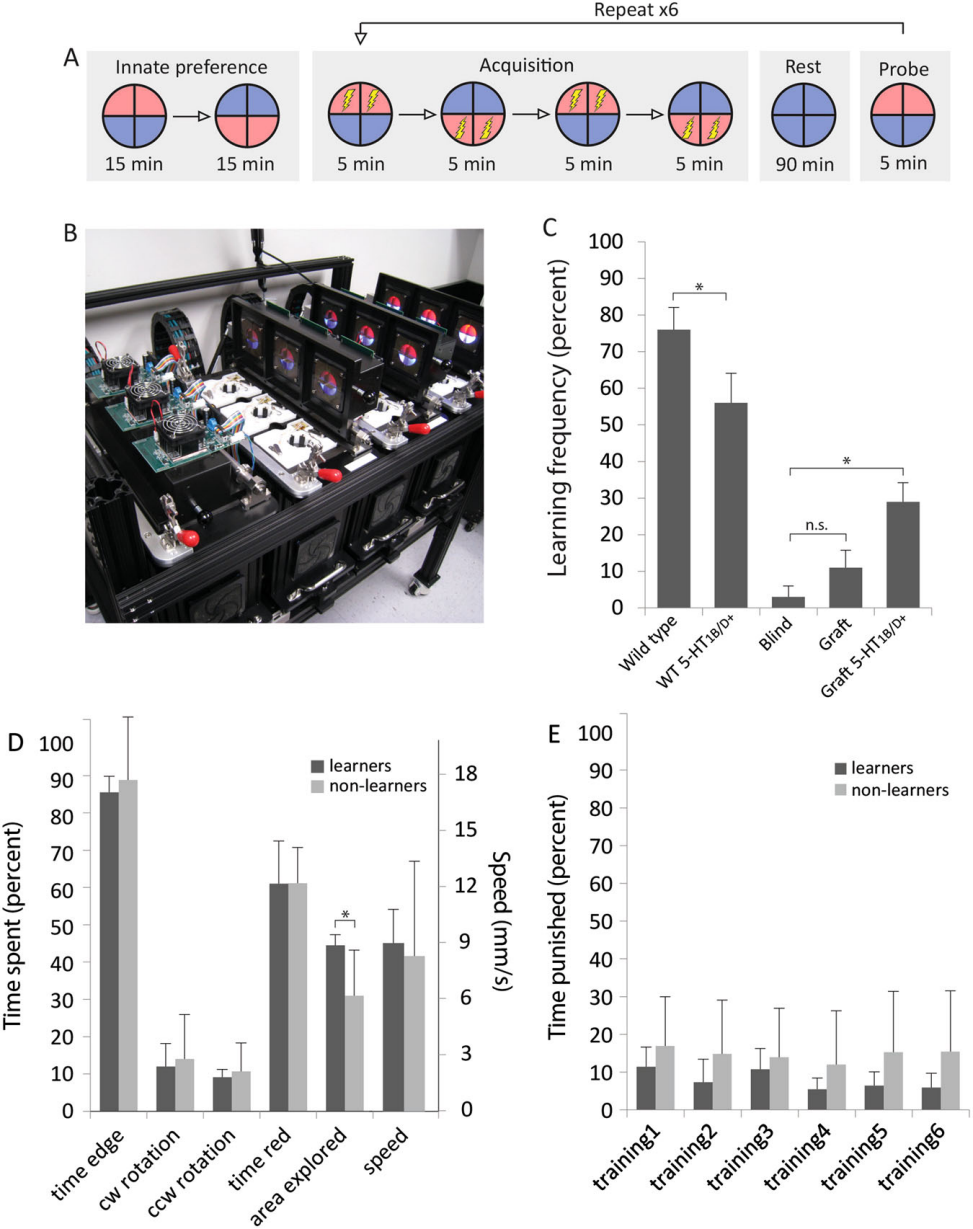
Eye grafts result in better performance in a visual assay when exposed to a 5-HT_1B/D_ activator. **a** The training regime used with stage 48–49 tadpoles consisted of an innate preference test, a training phase, a rest period, and finally a learning probe. **b** Training and testing was executed using an automated training device. Motion tracking cameras under each arena recorded tadpole position/behavior and software issued punishments according to the training parameters. **c** Wild-type animals learn with high frequency in the device, while few blind animals show learning. Animals with untreated eye grafts do not learn at a greater frequency than blind individuals, while those with innervated grafts in response to 5-HT_1B/D_ activation demonstrate significantly higher learning frequency than do blind animals. **d** When comparing basic behaviors between 5-HT_1B/D_ innervated learners and non-learners, the only significant difference detected was that animals that learned in the device explored more of the arena than those which did not. **e** Comparing the time punished during training for 5-HT_1B/D_ innervated animals reveals that learners receive significantly less punishments than individuals who do not learn in the device. *n* = 50, 38, 32, 43, and 24 for wild-type, wt + 5-HT_1B/D_, blind, ectopic eye, and ectopic eye + 5-HT_1B/D_, respectively. *n* = 8 for learners and 16 for non-learners. *Asterisk* indicates *p* ≤ 0.05. Values represent mean ± 1 standard deviation

To determine if blinded animals receiving eye grafts could learn in a visual assay and whether increased innervation leads to better performance, a custom automated behavior apparatus was employed. The device uses motion tracking cameras to record animals’ positions in an arena illuminated with red or blue light and issues commands to punish animals with a 1.2 mA current based on location. Briefly, the general training regime evaluates the animal’s preference for red or blue, trains the animal to avoid red by punishing it when it occupies a red quadrant, and examines any change in color preference after a rest periods (Fig. 5a, b). By repeating this procedure, a reproducible red-avoidance behavior is observed; this approach has been used to successfully train animals in multiple studies.^26, 27^

The majority of wild-type animals learn in our automated assay (Fig. 5c), with 76% of trained animals (*n* = 50) demonstrating learning, compared with blinded animals, which rarely meet the learning criteria (5%, *n* = 20). Non-blinded animals raised in 50 µM Zolmitriptan were also able to learn in the device, but at a reduced frequency compared with untreated siblings (*z* = 2.05, *p* = 0.02). The presence of an eye graft in blinded animals resulted in a learning frequency of 11%, a rate which was not significantly higher than non-grafted siblings (*n* = 43, *z* = −0.77, *p* = −0.221). However, in animals receiving grafts that innervated the host in response to 5-HT_1B/D_ activation, the frequency of learning was significantly higher compared with blinded animals (*n* = 24, *z* = −2.30, *p* = 0.011). Taken together, these results indicate that blinded animals fail to learn in an automated visual assay, but learning can be induced when animals receive eye grafts that were made to innervate the host by 5-HT_1B/D_ activation.

To better characterize the behavior of innervated graft animals that learned in the assay and those that did not, the following behaviors were compared between the two groups during across the innate portion of the trial: time spent at the edge vs. center of the arena, time spent swimming in a clockwise/counterclockwise direction, time spent in the red half of the dish, area of the dish explored, and speed (Fig. 5d). None of these behaviors differed between innervated learners and non-learners, except for exploration: animals that learned explored a greater portion of the dish than non-learning siblings (*t*-test, *p* = 0.024). These results were similar when comparing across treatments, as wild-type and blind controls showed minimal differences between basic behaviors in the device (Sup. Fig. 1). In addition to basic behavior, the proportion of time punished during each of the training periods was compared for innervated learners and non-learners, as poor performance during training could indicate a difficulty in perceiving the overhead lighting conditions. This analysis revealed a significant difference between groups (Fig. 5e), with non-learners receiving more punishments than learners in any given training session (two-way ANOVA, *p* = 0.004). In addition, punishments during training sessions tended to decrease among the animals that demonstrated learning, while non-learners trended upward across trials. These data support the learning results and indicate that the 5-HT_1B/D_-activated eye-graft animals that learned in the device were better able to discriminate the associative stimuli (color) during training sessions in the automated assay.

### Graft innervation confers image-forming vision: a moving pattern assay

**Fig. 6:**
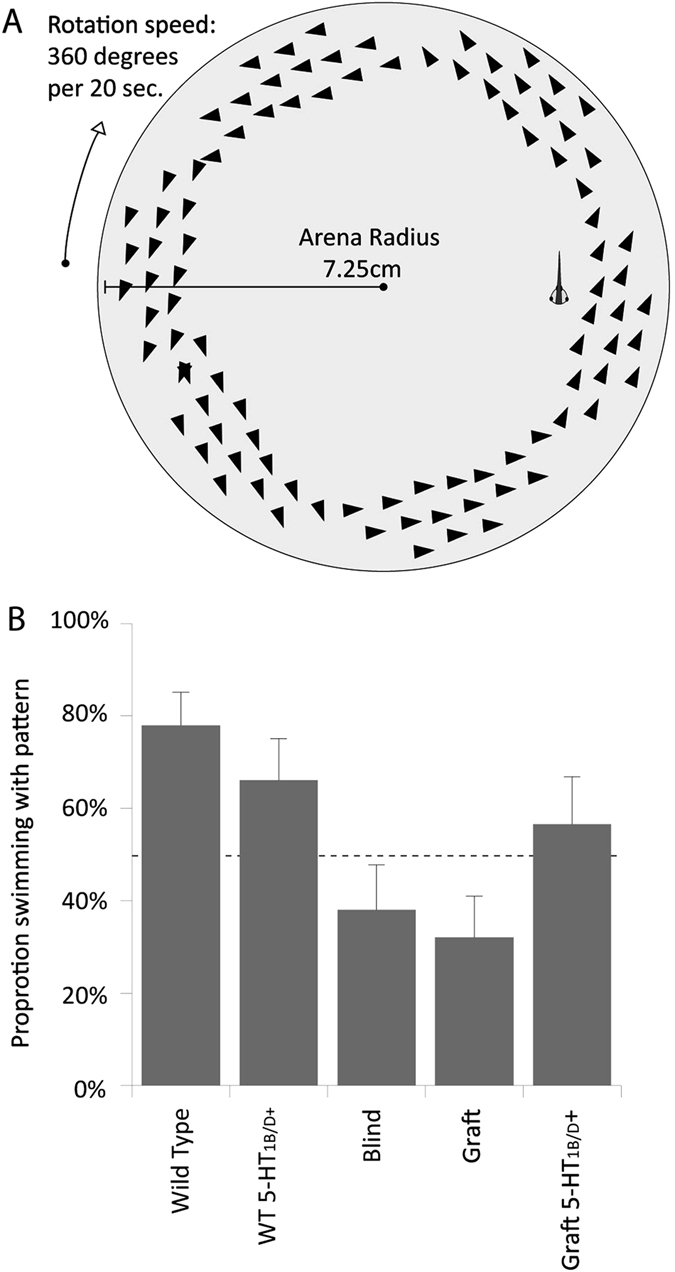
*Xenopus* tadpoles follow a rotating pattern presented from below. **a** Schematic of rotation trial in which dishes containing 3–5 tadpoles were placed above a monitor that displayed a rotating patterns of triangles. Animals were given 15 min of the pattern rotating in the clockwise direction followed by 15 min in the counterclockwise direction. Behavior was scored and averaged across both phases of the trial. **b** Wild-type animals perform well in the assay, with 80% of individuals correctly following the direction of rotation and wild-type animals raised in a 5-HT_1B/D_ agonist perform similarly. Blind animals and individuals with untreated eye grafts perform poorly, while animals with innervated grafts perform at intermediate levels. *χ* ^2^ = 18.76, *p* < 0.001. *n* = 34, 28, 25, 28, 23, for wild-type, wt + 5-HT_1B/D_, blind, ectopic eye, and 5-HT_1B/D_-activated, respectively. Values represent mean ± 1 standard deviation

In addition to wavelength discrimination, tadpoles were tested for true vision—the ability to follow patterns rotating clockwise and counterclockwise directions. The patterns were presented from below the tadpoles, and consisted of groups of triangular clusters rotating at a rate of 18° per second (Fig. 6a). Cohorts from each treatment were placed in dishes above the pattern, and their swimming direction as scored after 15 min of clockwise and counterclockwise pattern rotation—final performance scores were averaged between the two tests. Wild-type animals were able to follow the pattern well, with 80% of tested animals following the pattern after averaging the clockwise and counterclockwise portions of the trial (Fig. 6b). In comparison, blinded animals performed poorly, with only 38% of individuals swimming in the correct direction. Animals with an untreated ectopic eye also performed poorly, with 32% of tadpoles following the pattern. However, blinded animals with innervated grafts induced by exposure to the 5-HT_1B/D_ activator exhibited much better performance levels, with 57% of animals able to follow the rotating patterns. Analysis of the distribution revealed a significant effect of the treatment on the result compared with expected values (*χ* ^2^ = 18.76, *p* < 0.001). Taken together, these data reveal that animals receiving grafts in the presence of 5-HT_1B/D_ activation perform better in a pattern-movement task than those with non-treated grafts.

## Discussion

Previous work demonstrated that depolarization of resting potential in host tissue could be used to induce innervation of eye grafts in *Xenopus* tadpoles in a serotonin-dependent manner.^21^ Here, we examined the downstream pathway and showed that 5-HT_1B/D_ activation is sufficient to drive afferent innervation, even in the absence of bioelectric cues. Untreated grafts demonstrate very low rates of innervation, with less than 5% of grafts showing any neurites penetrating the host via fluorescent labeling. However, when grafted animals were reared in the 5-HT_1B/D_ activator Zolmitriptan, 40% of the transplants innervated the host, with afferent innervation present in the ventral fin and trunk of the tadpoles (an affect which only occurred when the eyes were placed at ectopic locations). Neurite extension could be observed 4 days post-transplant, a timing that is similar to that of untreated grafts or those which innervate in response to extracellular 5-HT exposure^24^; axons continued to arborize for the 5 days of imaging prior to behavior experiments.

The phenotype appeared to be specific to the eye, as other elements of the host CNS were unaffected by the presence of Zolmitriptan. Immunohistochemistry against anti-acetylated alpha tubulin allowed visualization of the lateral line, olfactory organs, and motor axons of the posterior fin, none of which were altered in treated animals. These results are similar to work in mouse, which have also found roles for serotonin in retinal ganglion axon guidance. Monoamine oxidase A knockout mice display improper segregation of retinal ganglion cells originating from the left and right eye,^14^ and similar to the present study 5-HT_1B/D_ activation has been shown to have effects on both retinal and CNS patterning.^13, 28, 29^ Conservation of mechanism across models suggest serotonin receptor activation/repression may be of future clinical use in medical stem cell therapies and transplant surgeries, helping tune retinal ganglion innervation in the host.

A possible mechanism by which 5-HT_1B/D_ activation leads to increased innervation can be proposed based on in vivo and in vitro data in mammalian systems. 5-HT_1B/D_ receptor binding initiates a number of physiological and metabolic changes in an expressing neuron including: the inhibition of neurotransmitter release, increased inositol phospholipid hydrolysis, opening of potassium channels, closure of voltage-gated calcium channels, and inhibition of adenylate cyclase activity. With regard to pathfinding, the latter mechanism has received the most attention, as cyclic nucleotide levels have been correlated with a number of neurite pathfinding behaviors. The effect of 5-HT_1B/D_ activation on mouse dorsal thalamus axons (conversion of netrin-1 attraction to repulsion) is inhibited in vitro by pharmacological activation of cyclic adenosine monophosphate (cAMP), while chemical reduction of cAMP recapitulates the effect even in the absence of 5-HT_1B/D_ activation.^13^ Dynamic cyclic nucleotide levels have been observed in a number of axon growth and pathfinding systems,^30^ and suite of recent fluorescent biosensors have been developed allowing for finer scale resolution of cAMP dynamics across neural development.^31^

Using an automated training assay, we demonstrated that blinded animals receiving eye grafts in combination with 5-HT_1B/D_ activation can learn wavelength-based cues with greater frequency than animals with untreated grafts. The underlying mechanism behind this observation is not yet clear, although in no cases were labeled neurons from the grafted eyes visualized within the brain of the host. Given this finding, we suggest that retinal ganglion cells from the graft do not connect to the host brain directly, but may pass information through other nerves in the spinal cord, and/or may interact with other sensory systems, such as those in the epidermis. This would be similar to data on human patients in studies of sensory substitution devices (including a number of well-characterized human–machine interfaces) such as cameras that deliver electrical stimuli to the skin or tongue of blind individuals.^1^ Use of these devices over extended periods of time leads to activation of the patients visual hemisphere, even though such machines are not directly connected to the users CNS.^32–35^ Dissecting the encoding and cellular paths for the ectopic eye-derived information, via whole-body neural imaging during active behavior, is a key area for future advances in this system.

Comparing the basic behaviors of animals that learned in the device to those that did not learn across the pre-training portion of the test did not reveal any notable differences in speed, time spent at the edge vs. center of the arena, clockwise vs. counterclockwise frequency, time spent in the red half of the dish, or area explored. This is perhaps unsurprising as wild-type and blind animals also did not show any large changes in these metrics: the only significant difference detected was that blind animals swim faster than untreated siblings. Given that the basic behaviors of all treatments receiving grafts are indistinguishable from wild-type animals, it is likely the presence of a posterior eye does not significantly alter swimming behavior in a way that would confound the learning results; when analyzing basic behavior, grafted animals move about the arena exactly as unoperated individuals.

Significant differences were detected between 5-HT_1B/D_-activated graft recipients when punishment was analyzed across training periods in the learning assay. Animals that were able to learn in the device received fewer punishments across all training sessions, which suggests they were better able to distinguish the wavelength cues present on both sides of the dishes. Further evidence for this hypothesis is that the frequency of punishments decreased by 50% between the first and last training session for animals that learned in the device, while the frequency remained constant for non-learners. This result provides further support that animals possessing innervated eye grafts in response to 5-HT_1B/D_ activation were able to use color as a cue in the associative learning assay.

In addition to color-learning data, we developed a novel test for image-forming vision: a rotation test pattern where tadpoles follow a rotating pattern of triangles presented from below via a LED monitor. Wild-type animals responded well in the assay, and the results corroborated earlier reports that *Xenopus* tadpoles tend to aggregate even when separated by physical barriers,^36^ which suggests the behavior is visually mediated. Anecdotal evidence suggested numerous reasons for grouping and aligning, from enhanced efficiency of fluid flow, aiding in filter feeding, to protection from predation via group effects; we hypothesize that the tadpoles are perceiving the triangles as conspecifics, but cannot rule out that they may identify the triangles themselves as food. Regardless, wild-type animals preferentially established a rotation direction corresponding to the rotating pattern below the dish. Blind animals performed poorly in the assay: individuals still spent the majority of their time swimming in circular patterns, but the direction was random with respect to the rotating display. Blinded animals receiving eye grafts in the presence of 5-HT_1B/D_ activation performed at intermediate levels to those of wild-type or blind animals without 5-HT_1B/D_ activation (significantly better than blinded animals in a pairwise comparison [*z* = 1.75, *p* = 0.040]), similar to the intermediate performance in the color-learning assay. This suggests that forcibly innervated grafts improve not only wavelength identification, but also the ability to resolve movement and directionally of external objects—a fairly sophisticated vision task.

Taken together, our study contributes a number of novel elements to the interdisciplinary science at the intersection of regenerative medicine, neuroscience, and developmental/evolutionary biology. We established a highly tractable model in which the anatomical and functional fate of transplanted organs can be studied in vivo. Remarkably, tadpoles can see via posterior eyes; the brain is not only plastic enough to make use of visual data no matter where in the body they originate, but can also do it when the data are communicated to the spinal cord. This suggests that biomedical implants may not need to interface to the brain but could instead provide function via connection to anatomical sites with lower surgery risk. *Xenopus* transplantation of eyes and other organs will facilitate the study of the mutual plasticity of the brain and body, as these larvae can be subjected to genetic, biophysical, pharmacological, and behavioral manipulations and assays. Understanding these processes will be crucial to the development of therapies ranging from individual stem cell implants to whole organ transplantation for somatic repair or even sensory enhancement/augmentation. We identified a serotonergic compound that can be used to control the extent of nerve growth in vivo and are currently testing it in mammalian bioengineering contexts in vitro. Given the increasing recognition of neurotransmitter functions in developmental contexts outside the brain, it is likely that neurotransmitter-targeting compounds will be increasingly exploited as an exciting toolkit for modulation of form and function in vivo. Our identification of a human-approved drug that drastically increases innervation between the host and implant, and improves behavioral function, suggests numerous applications. We envision a roadmap that exploits the innate plasticity and robustness of embodied neural systems for advances in regenerative therapies.

## Materials and methods

### Animal husbandry

*Xenopus* tadpoles were reared in 0.1× Marc’s Modified Ringers solution (MMR), pH 7.8, under standard procedures^37^ and were staged according to Neiuwkoop and Faber.^38^ All embryos (pooled from separate mothers then randomly divided between treatments) were placed at 22 °C overnight, to achieve the correct stages for microsurgery, and were then moved to 16 °C for the remainder of the experiment. For animals used in behavioral trials, individuals were raised under a 12 h:12 h light:dark cycle in a 100 × 25 mm petri dish at a density of no more than 30 individuals per dish. Upon reaching swimming stages, animals were fed 5 days a week, M-F, once a day in the evening with Sierra Micron powdered diet, and water changes were performed M/W/F. All experimental procedures using animals for experimental purposes were approved by the Institutional Animal Care and Use Committee and Tufts University Department of Laboratory Animal Medicine under protocol number M2014-79.

### Microinjection

Labeled fluorescent donor embryos were created through microinjection of tdTomato mRNA. All mRNA was synthesized using standard message machine kits (Life Technologies) and stored at −80 °C until used. Embryos were transferred to 3% Ficoll solution before being microinjection with a pulled capillary, which delivered approximately 550 ng of RNA in a 50 nl volume to each of four cells at the three-cell stage (to maximize expression). Two hours after microinjection, embryos were washed twice in 0.1× MMR and moved to a 22 °C incubator overnight. The following morning, each embryo was screened for fluorescence using an Olympus BX-61 microscope equipped with a Hamamatsu ORCA AG CCD camera, and the animals with the most intense signal were separated to be used as graft donors. This step was necessary as we found tdTomato expression to be more mosaic among animals than other fluorescent reporters.

### Microsurgery

Prior to microsurgery, the vitelline membranes of stage 23 tdTomato donors and wild-type recipients were removed and the embryos were anesthetized in 0.02% tricaine solution, pH 7.5, in 0.1× MMR. Using surgical forceps, the developing eye primordia of tdTomato embryos was excised, taking care to remove as little underlying tissue as possible. Then, a small slit was placed in the posterior of wild-type recipients, just ventral to the neural tube, by puncturing the tissue with the forceps in the closed position and then opening them slightly to expand the wound for graft implantation. Once the recipient was prepped, the tdTomato eye primordium was carefully lifted by hand and placed into the wild-type recipient at the wound location. Care was made to orient the graft with the appropriate proximal/distal orientation (i.e., with the eye facing “out”), although tissue could be rotated along its radial axis. Grafts fused with the host within 10 min and animals were given 30 min to heal before being washed in 0.1× MMR and moved to a 16 °C incubator. In addition, to avoid any possible confounding effects of laterality, only the left eye of the donor was removed, and all grafts were placed on the left side of the recipient embryo. For blinded animals used as controls, stage 34 animals were anesthetized in 0.02% tricaine for 30 min. Using surgical forceps, a cut was made in the epidermis just dorsal to the eye, and the entire ocular structure was removed. Animals were allowed to heal for 30 min before being washed twice in 0.1× MMR and returned to the 16 °C incubator until behavior testing.

### Pharmaceutical exposure

For animals exposed to the 5-HT_1B/D_ activator Zolmitriptan (Sigma SML0248), 50 mM stocks were prepared in DMSO and frozen at −20 °C until used. Once operated animals were given 30 min to heal, they were moved to new dishes containing 50 µM Zolmitriptan in 0.1× MMR. Pharmaceuticals were refreshed every other day and removed at stage 46, after which all animals were reared in standard 0.1× MMR.

### Immunohistochemistry

Host innervation was visualized through immunohistochemistry with the monoclonal anti-acetylated alpha tubulin antibody (Sigma T7451) using a previously described protocol.^39^ Briefly, animals were anesthetized in 0.02% tricaine for 30 min, then fixed for 2 h at room temperature in MEMFA (100 mM MOPS (pH 7.4), 2 mM EGTA, 1 mM MgSO_4_, 3.7% (v/v) formaldehyde). Following fixation, animals were washed three times, 10 min per wash, in phosphate buffered saline + Tween 20 (PBST), and then blocked for 1 h at room temperature with 10% goat serum in PBST. Samples were then rocked overnight at 4 °C in monoclonal anti-acetylated alpha tubulin antibody, diluted 1:500 in PBST + 10% goat serum. Following primary exposure, samples were washed three times for 15 min in PBST before a 60 min secondary incubation with AlexaFlour-555 conjugated secondary at 1:1000 diluted in PBST. Following secondary incubation, samples were washed three times for 15 min in PBST and imaged on an Olympus BX-61 microscope.

### Behavior testing

All wavelength-learning trials were performed with a custom built automated training system.^26, 40^ The device consists of an array of 12 individual chambers, each containing a disposable 60 × 15 mm petri dish filled with 15 ml of 0.1× MMR. Below each dish is a machine vision camera (Insight-Micro 1400, Cognex Corporation, Natick, MA, USA), which uses a firmware-embedded background subtraction algorithm to plot the location of any animals in the chamber in a Cartesian manner. Illumination can be provided to each chamber independently from above, by an illumination control module which can specify color and intensity by quadrant. Red or blue light is delivered by light-emitting diodes (Osram Semiconductors, blue LED; 470 nm part no. LBW5SM, red LED, 635 nm part no. LRG6SP) and *Xenopus* are expected to see both given the spectral profiles of their three known cone classes.^41^ Finally, within each chamber is also a set of six iridium oxide-coated titanium electrodes allowing the delivery of mild to strong electric shocks. All shocks delivered during wavelength-mediated training experiments were 1.2 mA AC currents, pulsed for 100 ms followed by 300 ms of no shock. This value was previously determined to be the lowest that elicits a behavioral response and no animals displayed physiological or behavioral abnormalities upon completion of testing.

To determine if tadpoles could learn in a color-based associative assay, the following trial was performed^26^: individuals were first presented with an arena which was illuminated half with red light, half with blue light, in the absence of any punishment to probe innate color preferences. Innate testing lasted 30 min and the position of the colors was inverted after 15 min to avoid having stationary tadpoles be scored as having a 100% preference for either color (inverting the lights would result in a 50/50 preference). After innate testing, tadpoles enter the learning acquisition phase, where individuals are again presented with red and blue halves of the dish, but in this case the tadpole receives a 1.2 mA shock if it occupies a red quadrant. Acquisition duration is 20 min, with the colors in the chamber being inverted every 5 min. Following acquisition, tadpoles are given a 90-min rest in which the entire chamber is illuminated with blue light, and no punishment is delivered. Finally, individuals are probed for learning by giving them a choice between red and blue for 5 min, with neither sample receiving punishment. The entire block of acquisition-rest-probe is repeated six times. All tadpoles used in learning trials were stage 48, and an individual was determined to have learned if their preference for red was below 30%, averaged across the final three probes of the experiment. Further, tadpoles were fed directly before associative learning experiments, and food was added to each arena during training, as hungry animals fail to learn in the assay. Animal location was varied across trials to ensure treatments were evenly distributed between chambers, avoiding any confounding effect of position.

In addition to red/blue wavelength discrimination, tadpoles were tested to determine whether they could respond to the movement of patterns on a white background. For this assay, 3–5 tadpoles were tested simultaneously in a 15-cm diameter petri dish filled with 0.1× MMR. Dishes were placed on top of a LCD monitor screen (model Lenovo D221), and animated patterns were displayed under the bottom of the dish. The animated pattern consisted of groups of triangles around the outer portion of the dish, which would rotate in a clockwise or counterclockwise direction at a speed of 1 revolution per 20 sec (or 18°/s)—the approximate speed of a stage 48 tadpole swimming around the edge of the dish. Tadpoles were placed in the dish for 15 min with the pattern rotating clockwise, after which their direction of swimming was scored [with the possible outcomes being clockwise, counterclockwise, or other—which included no movement, traversing the center of the dish (defined as any open area not covered by the pattern), or colliding with another animal]. After the clockwise test, the rotation of the pattern was set to counterclockwise and the tadpole swimming direction was scored again after 15 min had passed. For all pattern movement trials, food was withheld for 24 h pre-experiment, as satiated animals often did not swim often enough to score directionality.

### Imaging

To image innervation by implanted eye grafts, stage 46 animals were anesthetized for 10 min in 0.02% tricaine solution, pH 7.5, and transferred to depression slides. Donor fluorescence was imaged with an Olympus BX-61 microscope using a TRITC filter set, which was then overlaid with bright field images. Individuals were imaged at 40× and 100× magnifications, and widefield stacks were used to resolve the region of interests. Host innervation was imaged following immunohistochemistry using the same Olympus BX-61 and TRITC filter set.

### Statistics

Statistical analyses were performed using Prism v.5 (GraphPad Software, La Jolla, CA, USA). Animal numbers were chosen based on previous studies as well as sample size estimates given a type I error rate of 5% and power of 0.8. Host innervation data were collected as binomial outputs (presence or absence of innervation phenotype) and analyzed using non-parametric statistics. For color-learning assays, animals were considered to have learned if their average preference over the last three testing periods was equal to or below 30%. Results were then compared using a two proportion *z*-test, with Bonferroni corrections to adjust experiment-wide *α* level, where multiple comparisons were made within an experiment. Comparison of punishment between tadpole learners and non-leaners was performed using a two-way ANOVA.

## Electronic supplementary material

Supplemental Fig. 1

## References

[1] Bach-y-Rita P, S WK (2003). Sensory substitution and the human-machine interface. Trends Cogn. Sci..

[2] Bach-y-Rita, P. *Brain Plasticity*. 113-118 (Mosby, 1988).

[3] Arno P (2001). Occipital activation by pattern recognition in the early blind using auditory substitution for vision. Neuroimage.

[4] De Volder AG (1999). Changes in occipital cortex activity in early blind humans using a sensory substitution device. Brain Res..

[5] Bach-y-Rita, P. *Nonsynapctic Diffusion Neurotransmission and Late Brain Reorganization* (Demos-Vermande, 1995).

[6] De Volder AG (2001). Auditory triggered mental imagery of shape involves visual association areas in early blind humans. Neuroimage.

[7] Meijer PB (1992). An experimental system for auditory image representations. IEEE Trans. Biomed. Eng..

[8] Tyler M, Danilov Y, Bach YRP (2003). Closing an open-loop control system: vestibular substitution through the tongue. J. Integr. Neurosci..

[9] Karcher SM, Fenzlaff S, Hartmann D, Nagel SK, Konig P (2012). Sensory augmentation for the blind. Front. Hum. Neurosci..

[10] Nakano T (2012). Self-formation of optic cups and storable stratified neural retina from human ESCs. Cell Stem Cell.

[11] Eiraku M (2011). Self-organizing optic-cup morphogenesis in three-dimensional culture. Nature.

[12] Haydon PG, McCobb DP, Kater SB (1987). The regulation of neurite outgrowth, growth cone motility, and electrical synaptogenesis by serotonin. J. Neurobiol..

[13] Bonnin A, Torii M, Wang L, Rakic P, Levitt P (2007). Serotonin modulates the response of embryonic thalamocortical axons to netrin-1. Nat. Neurosci..

[14] Upton AL (1999). Excess of serotonin (5-HT) alters the segregation of ispilateral and contralateral retinal projections in monoamine oxidase A knock-out mice: possible role of 5-HT uptake in retinal ganglion cells during development. J. Neurosci..

[15] Elliott KL, Fritzsch B (2010). Transplantation of *Xenopus laevis* ears reveals the ability to form afferent and efferent connections with the spinal cord. Int. J. Dev. Biol..

[16] Elliott KL, Houston DW, Fritzsch B (2013). Transplantation of *Xenopus laevis* tissues to determine the ability of motor neurons to acquire a novel target. PLoS ONE.

[17] Elliott KL, Houston DW, Fritzsch B (2015). Sensory afferent segregation in three-eared frogs resemble the dominance columns observed in three-eyed frogs. Sci. Rep..

[18] Constantine-Paton M, Capranica RR (1976). Axonal guidance of developing optic nerves in the frog. II. Electrophysiological studies of the projection from transplanted eye primordia. J. Comp. Neurol..

[19] Constantine-Paton M, Capranica RR (1976). Axonal guidance of developing optic nerves in the frog. I. Anatomy of the projection from transplanted eye primordia. J. Comp. Neurol..

[20] Constantine-Paton M, Caprianica RR (1975). Central projection of optic tract from translocated eyes in the leopard frog (*Rana pipiens*). Science.

[21] Blackiston DJ, Levin M (2013). Ectopic eyes outside the head in *Xenopus* tadpoles provide sensory data for light-mediated learning. J. Exp. Biol..

[22] Levin M (2014). Molecular bioelectricity: how endogenous voltage potentials control cell behavior and instruct pattern regulation in vivo. Mol. Biol. Cell.

[23] Levin M (2013). Reprogramming cells and tissue patterning via bioelectrical pathways: molecular mechanisms and biomedical opportunities. Wiley Interdiscip. Rev. Syst. Biol. Med..

[24] Blackiston DJ, Anderson GM, Rahman N, Bieck C, Levin M (2015). A novel method for inducing nerve growth via modulation of host resting potential: gap junction-mediated and serotonergic signaling mechanisms. Neurotherapeutics.

[25] Blackiston D, Adams DS, Lemire JM, Lobikin M, Levin M (2011). Transmembrane potential of GlyCl-expressing instructor cells induces a neoplastic-like conversion of melanocytes via a serotonergic pathway. Dis. Model Mech..

[26] Blackiston, D. J. & Levin, M. Aversive training methods in *Xenopus laevis*: general principles. *Cold Spring Harb. Protoc.* **2012**, doi:10.1101/pdb.top068338 (2012). 10.1101/pdb.top068338 PMC3617481 22550289

[27] Blackiston DJ, Levin M (2013). Inversion of left-right asymmetry alters performance of *Xenopus* tadpoles in nonlateralized cognitive tasks. Anim. Behav..

[28] Salichon N (2001). Excessive activation of serotonin (5-HT) 1B receptors disrupts the formation of sensory maps in monoamine oxidase a and 5-HT transporter knock-out mice. J. Neurosci..

[29] Upton AL (2002). Lack of 5-HT(1B) receptor and of serotonin transporter have different effects on the segregation of retinal axons in the lateral geniculate nucleus compared to the superior colliculus. Neuroscience.

[30] Piper M, van Horck F, Holt C (2007). The role of cyclic nucleotides in axon guidance. Adv. Exp. Med. Biol..

[31] Gorshkov K, Zhang J (2014). Visualization of cyclic nucleotide dynamics in neurons. Front. Cell. Neurosci..

[32] De Volder AG (1999). Changes in occipital cortex activity in early blind humans using a sensory substitution device. Brain Res..

[33] Dobelle WH (2000). Artificial vision for the blind by connecting a television camera to the visual cortex. ASAIO J..

[34] Humayun MS (2003). Visual perception in a blind subject with a chronic microelectronic retinal prosthesis. Vision Res..

[35] Ptito M, Moesgaard SM, Gjedde A, Kupers R (2005). Cross-modal plasticity revealed by electrotactile stimulation of the tongue in the congenitally blind. Brain.

[36] Wassersug RJ, Hessler CM (1971). Tadpole behaviour: aggregation in larval *Xenopus laevis*. Anim. Behav..

[37] Sive, H. L., Grainger, R. M. & Harland, R. M. *Early Development of Xenopus laevis* (Cold Spring Harbor Laboratory Press, 2000).

[38] Nieuwkoop, P. D. & Faber, J. *Normal Table of Xenopus laevis (Daudin)*. (North-Holland Publishing Company, 1967).

[39] Blackiston, D., Vandenberg, L. N. & Levin, M. High-throughput *Xenopus laevis* immunohistochemistry using agarose sections. *Cold Spring Harb. Protoc.* **2010**, doi:10.1101/pdb.prot5532 (2010). 10.1101/pdb.prot5532 PMC3654656 21123419

[40] Blackiston D, Shomrat T, Nicolas CL, Granata C, Levin M (2010). A second-generation device for automated training and quantitative behavior analyses of molecularly-tractable model organisms. PLoS ONE.

[41] Rohlich P, Szel A (2000). Photoreceptor cells in the *Xenopus* retina. Microsc. Res. Tech..

