# Supplementary figures and images for: Serotonergic stimulation induces nerve growth and promotes visual learning via posterior eye grafts in a vertebrate model of induced sensory plasticity
Source: NPJ Regen Med. 2017 Mar 30;2:8. doi: 10.1038/s41536-017-0012-5 (PMC5665622; doi:10.1038/s41536-017-0012-5)

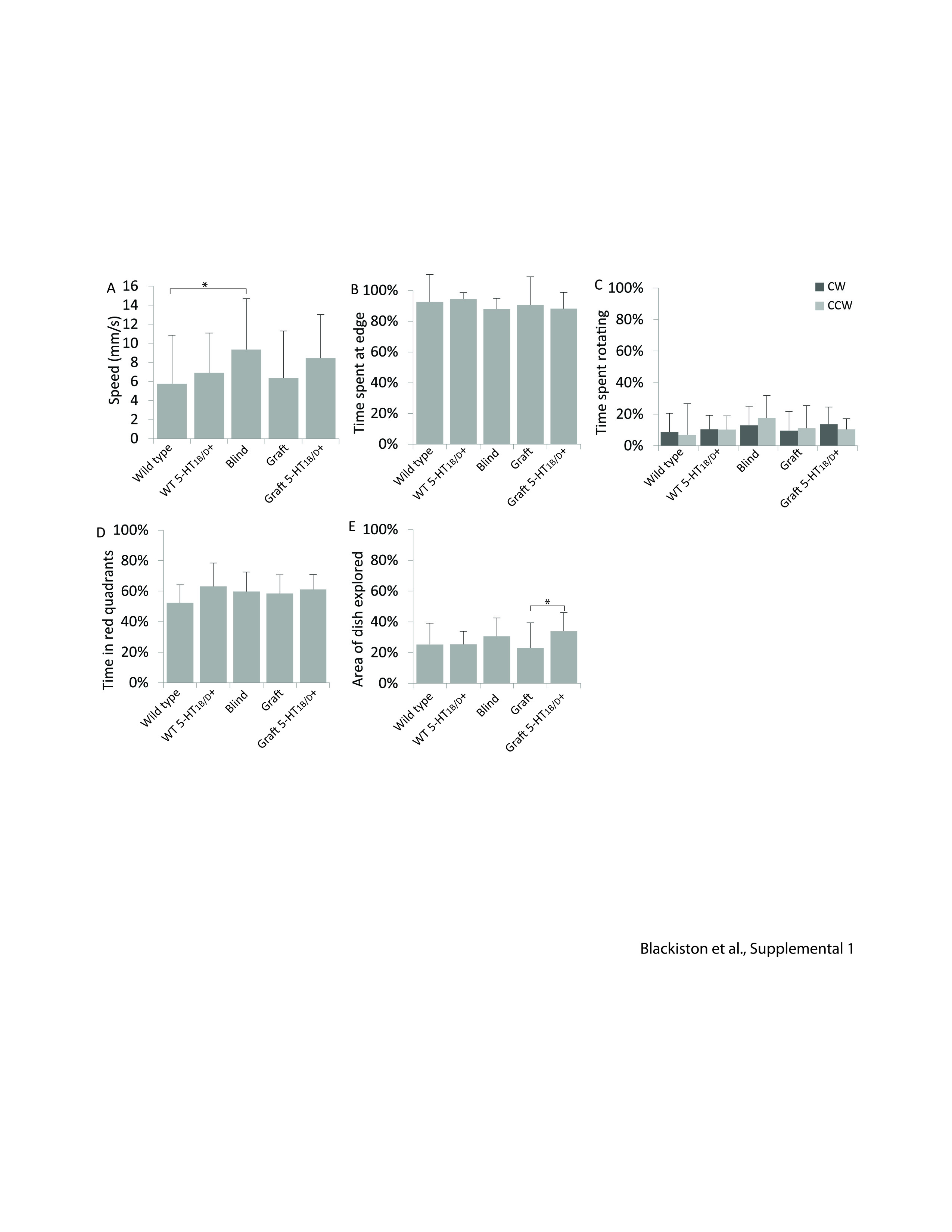

Supplement: Supplementary file 1 — Supplemental Fig. 1 [file 41536_2017_12_MOESM1_ESM.jpg]
